# Comparison of clinical bracket point registration with 3D laser scanner and coordinate measuring machine

**DOI:** 10.1590/2176-9451.20.1.059-065.oar

**Published:** 2015

**Authors:** Mahtab Nouri, Arash Farzan, Ali Reza Akbarzadeh Baghban, Reza Massudi

**Affiliations:** 1 Associate professor, Dentofacial Deformities Research Center of Shahid Beheshti University of Medical Sciences, Iran; 2 Postgraduate student of Orthodontics, Research Center of Shahid Beheshti University of Medical Sciences, Iran; 3 Assistant professor of Biostatistics, Faculty of Paramedicine, Shahid Beheshti, University of Medical Sciences, Iran; 4 Professor, Laser and Plasma Research Institute, Shahid Beheshti University, Iran

**Keywords:** Laser, Orthodontics, Computer-assisted image processing

## Abstract

**OBJECTIVE:**

The aim of the present study was to assess the diagnostic value of a laser scanner developed to determine the coordinates of clinical bracket points and to compare with the results of a coordinate measuring machine (CMM).

**METHODS:**

This diagnostic experimental study was conducted on maxillary and mandibular orthodontic study casts of 18 adults with normal Class I occlusion. First, the coordinates of the bracket points were measured on all casts by a CMM. Then, the three-dimensional coordinates (X, Y, Z) of the bracket points were measured on the same casts by a 3D laser scanner designed at Shahid Beheshti University, Tehran, Iran. The validity and reliability of each system were assessed by means of intraclass correlation coefficient (ICC) and Dahlberg's formula.

**RESULTS:**

The difference between the mean dimension and the actual value for the CMM was 0.0066 mm. (95% CI: 69.98340, 69.99140). The mean difference for the laser scanner was 0.107 ± 0.133 mm (95% CI: -0.002, 0.24). In each method, differences were not significant. The ICC comparing the two methods was 0.998 for the X coordinate, and 0.996 for the Y coordinate; the mean difference for coordinates recorded in the entire arch and for each tooth was 0.616 mm.

**CONCLUSION:**

The accuracy of clinical bracket point coordinates measured by the laser scanner was equal to that of CMM. The mean difference in measurements was within the range of operator errors.

## Abstract (pt)

**OBJETIVO:**

o objetivo do presente estudo foi avaliar o valor diagnóstico de um *scanner* a *laser* desenvolvido para determinar as coordenadas dos pontos de colagem de braquetes, comparando seus resultados aos resultados obtidos com uma máquina de medição coordenada (MMC).

**MÉTODOS:**

esse estudo experimental diagnóstico foi conduzido com modelos ortodônticos obtidos a partir da arcada superior de 18 pacientes adultos, com oclusão normal de Classe I. Inicialmente, as coordenadas dos pontos de colagem de braquetes de todos os modelos foram mensuradas por uma MMC. Em seguida, as coordenadas tridimensionais (X, Y, Z) dos pontos foram mensuradas nos mesmos modelos por um *scanner* a *laser* 3D, desenvolvido na Universidade de Shahid Beheshti. A eficácia e confiabilidade dos dois sistemas foram avaliadas pelo Coeficiente de Correlação Intraclasse (CCI) e pela fórmula de Dahlberg.

**RESULTADOS:**

a diferença entre a média da dimensão mensurada pela MMC e o valor real obtido foi de 0,0066mm (IC 95%: 69,98340 - 69,99140). A diferença média para o *scanner* a *laser* foi de 0,107 ± 0,133 (95% IC: -0,002 - 0,24). Em cada método, as diferenças não foram significativas. Ao comparar os dois métodos, o CCI gerou um valor de 0,998 para a coordenada X e de 0,996 para a coordenada Y. A diferença média para as coordenadas registradas em cada dente da arcada foi de 0,616mm.

**CONCLUSÃO:**

a precisão das coordenadas do ponto de colagem dos braquetes foi a mesma no *scanner* a *laser* e na MMC. A diferença média entre as medições manteve-se dentro dos limites de erros operacionais.

## INTRODUCTION

In order to prevent relapse during the retention period, it is paramount that the arch form be maintained. Therefore, before orthodontic treatment onset, patient's initial arch form should be determined and wires with the same arch form should be used throughout treatment so as to ensure stability of treatment results.

Various landmarks and tools have been used to assess patient's arch form. In previous studies, the midpoint of incisal edges and buccal cusp tips have been used as landmarks.^1^^,^ 2 However, with the technological advances in three-dimensional devices, buccal landmarks at bracket attachment points became available to be used for this purpose.^3^^-^ 6 This new technique helps in generating a more precise arch form, especially at force application points.

Various imaging techniques, such as radiography, photocopy, two-dimensional scanning,^5^ three-dimensional scanning^5^ and coordinate measuring machine (CMM),^7^ have been used to determine patient's dental arch form.

CMM is found to be the most accurate device for this purpose. Due to its mechanical nature and the presence of a touch probe, this technique has a high precision of approximately 10 µm and can be considered as the gold standard.^7^ Stereophotogrammetry and CBCT have also been introduced for 3D imaging with the use of laser or regular light. Of the mentioned techniques, laser scanner is found to be an accurate method. OraScanner, for instance, was reported to have an accuracy of approximately 30-50 µm.^8^ The voxel size in CBCT is of approximately 0.125 mm.^9^

After determining the landmarks with an accurate imaging technique, a mathematical model is adopted to these points, following a straight curve to be used in straight wire techniques. Currently, second and third order bends can be performed by the use of robotics; however, these methods have not gained much popularity due to the complexity and high costs of the technique. Although different mathematical models, such as the fourth-degree polynomial equation, beta-function and cubic spline, have been used in different studies, mostly, the use of polynomial equation has been suggested.^10^^-^ 18

In Iran, as in other Middle Eastern countries, the use of these technologies is not feasible, since the majority of companies do not operate in this area. Therefore, we developed a laser scanner as well as its associated software to generate arch form using a fourth-degree polynomial equation. The scanner was developed at the Orthodontics and Dentofacial Orthopedics Department of Shahid Beheshti Medical University.

The aim of the present study is to assess the diagnostic value of this laser scanner designed to determine the coordinates of clinical bracket points, and to compare the results with the results yielded by CMM.

## MATERIAL AND METHODS

This diagnostic experimental study was conducted on maxillary and mandibular orthodontic study casts of 18 adults with normal Class I occlusion and fully erupted permanent teeth including second molars. Patients did not have crowding or midline shift and teeth had no abrasion, fracture, or ectopic eruption.

**Figure 1 -:**
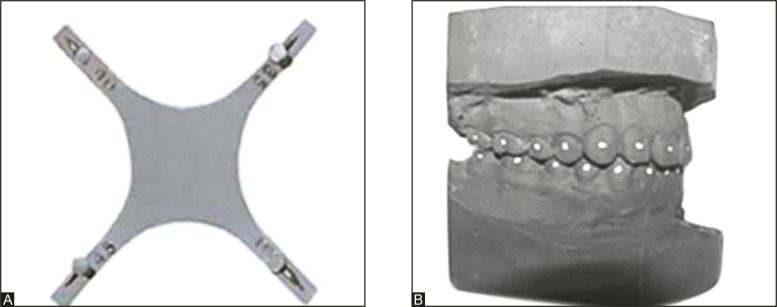
Preparation of dental cast for digitization by CMM and laser scanner. A) Orthodontic measuring gauge used to determine CBPs. B) Final dental casts after being painted and marked for CBPs.

In order to create maximum contrast for visual detection, all casts were colored black, using water-soluble dye (Pars Co., Tehran, Iran) and a brush. Afterwards, clinical bracket points were marked on each tooth according to the bracket placement guide for prefabricated appliances.^19^ An orthodontic gauge (Unitek, USA) and a fine tip white nail polish measuring 2 mm in diameter (Nail Design Polish, Victoria, Taiwan, Taiwan) were used (Figs 1A-C).

**Figure 2 -:**
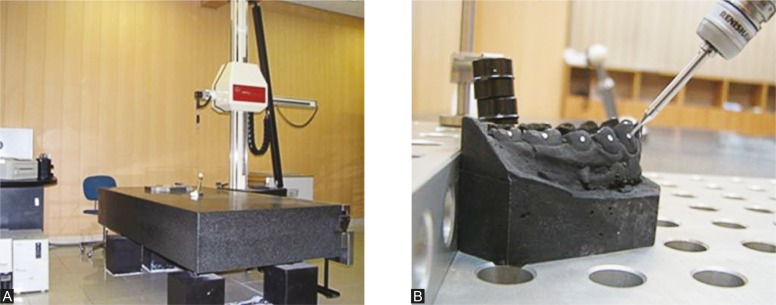
Coordinate measuring machine (CMM). A) Device setting general view. B) Dental casts are placed to have CBPs digitized by the touch probe of the CMM.

In the first part of the study, the coordinates of bracket points were measured on all casts by a coordinate measuring machine (CMM) (Mora, Aschaffenburg, Germany) with 10 ± 0.01 micrometer precision. Files were digitally saved in .txt format (Figs 2 A,B). This device has a touch probe with a diameter of 2 mm. When the operator touched the respective point with the probe, the machine read the input from the probe and recorded the X, Y and Z coordinates of the point. In the second part of the study, the three-dimensional coordinates (X, Y, Z) of bracket points were measured on the same casts by a 3D laser scanner designed at Shahid Beheshti University, Tehran, Iran.^20^ Files were also saved in .txt format. The scanner consisted of two class 2 laser diodes and two charge-coupled devices (CCD) used to capture and transfer images into a computer.

## Scanner

**Figure 3 -:**
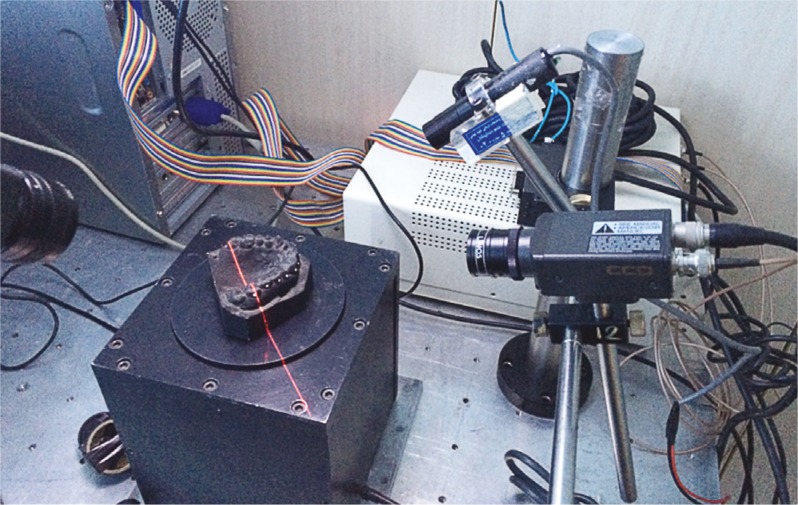
Dental casts are placed on the rotor for CBP digitization. Laser beam is irradiated onto the cast while it is rotated by the rotor.

Scanning was carried out with a 3D surface laser scanner. Our scanner^20^ consisted of two class 2 laser diodes operating at 685 nm with output power of 1 mW. Each laser produced a line 100-mm thick at a distance of 180 mm from the laser. Two CCDs (768 x 493 pixels, Hitachi KPM1, Japan) captured and transferred the image of the cast into a computer. The distance between the cameras and the object varied from 12 to 26 cm. The maximum area of test scanning was 6 x 6 cm^2^. The cast was secured to the horizontal surface of a rotating table controlled by a step motor which rotated the table with an accuracy of 0.009 degrees (Figs 3 A,B). To calibrate the scanner system, we attached two metal backing plates separating the rectangular grids (16 x 16 cm) with circles printed on paper. The diameter of each circle was 6 mm and the distance between them was 12 mm. The grid had an angle of 30 degrees relative to the side of the rectangle. The vertical distance between the two plates was 20 mm. For calibration, the grids were placed on the rotating table and the CCDs were adjusted so that the whole grid pattern could be imaged. Each CCD captured an image and the software merged both images into a final image. A program written in Visual Basic 6 environment was used to calculate the relative position of different points on the cast. To acquire such position, we first determined the location of the CCD and the laser relative to a specified point on the rotating table. Next, the cast, marked with a point painted on its surface, was adjusted on the rotating table. Having the resolution of the stepper motor and considering different reflections of the laser from the white color points on the cast, one can determine the position of those points in relation to the center of the coordinate.

## DATA ANALYSIS

## Reproducibility and Validity

Normally, the validity of CMM is annually controlled by the manufacturer. Additionally, a certificate of validity is issued. However, in this study, validity and reproducibility of the device were ensured by measuring the diameter of a reference metal master disc (Gauge disc, Mitutoyo, Osaka, Japan) with a known diameter of 69.994 mm at 20 °C. Measurements were taken by the operator for 10 times with at least one-day interval between each measurement. To assess reproducibility of the 3D laser scanner, a Teflon cube with dimensions of 31.90 x 31.90 mm was scanned. The values obtained were compared with actual dimensions of the cube. The dimensional measurements were exactly the same as the actual dimensions of the cube.

To assess the laser scanner validity in measuring clinical bracket point coordinates, the Y and X coordinates recorded on each cast were compared using the Y and X coordinates obtained from CMM readings as reference. Since the Z coordinate is not required for drawing dental arch curve, this coordinate was considered as zero for each point. To assess reproducibility of CMM, CMM measurements of the reference master gauge disc were compared with the actual measurements of the disc at 20 °C. To assess reproducibility of the laser scanner, CMM measurements of the Teflon cube were compared with 3D scanner measurements of the cube. The magnitudes of errors were determined by means, standard deviation and 95% confidence intervals. To assess the laser scanner validity in measuring the coordinates of clinical bracket points, the coordinates recorded by the laser scanner were compared with CMM reading using ICC. The numerical value of this error was calculated for each group of maxillary and mandibular teeth and compared with CMM measurements using Dahlberg's^21^ formula as the reference.

## RESULTS

The results of CMM reproducibility testing that included measurements of the diameter of a reference master gauge disc (Mitutoyo, Osaka, Japan) with a known diameter of 69.994 mm at 20 °C measured 10 times by the same operator showed that the mean recorded value was 69.98740 mm, with a range of 0.004 mm and standard deviation of 0.016 mm. The difference between the measured mean dimension and the actual value was 0.0066 mm. At 95% CI, this difference was not statistically significant.

Comparisons of the 10 measurements of the cube are presented inTable 1. The mean difference is 0.107 ± 0.133 mm (95% CI: -0.002, 0.24). Since zero falls within the confidence interval, there was no statistically significant difference between the two methods used to calculate the dimensions of the cube.

**Table 1 -:** Ten measurements of the Teflon cube.

Scanner measurements	31.92	32.15	32.07	32.11	32.01	31.79	32.17	32.05	31.80	32.01
Difference*	0.02	0.25	0.17	0.21	0.11	-0.11	0.27	0.15	-0.10	0.11

* Scanner value - reference value (31.90 mm).

ICC was 0.998 for the X coordinate and 0.996 for the Y coordinate; which were indicative of a very high similarity between measurements yielded by both methods: CMM and laser scanner. The numerical differences for the X and Y coordinates, according to Dahlberg's formula applied to various areas of the dental arch, are demonstrated inTable 2. It was the least for central incisors and the greatest for molars. The numerical differences in the X and Y coordinates of incisors were 0.345 and 0.426, respectively. The numerical differences in the X and Y coordinates of canines were 0.661 and 0.606, respectively. Also, the numerical differences in the X and Y coordinates of posterior teeth were 0.860 and 0.817, respectively. The greater the convexity of the tooth surface, the greater the difference between measurements. Thus, the maximum error value is usually observed in molars and first premolars. There was no difference between maxillary and mandibular measurements.

**Table 2 -:** Numerical differences for the X and Y coordinates according to Dahlberg's formula

X centrals	Y centrals	X laterals	Y laterals	X canines	Y canines	X premolars	Y premolars	X molars	Y molars	
0.252	0.365	0.439	0.487	0.661	0.606	0.776	0.695	0.945	0.939	Total
0.285	0.368	0.36	0.400	0.655	0.466	0.749	0.580	0.953	0.924	Upper arch
0.218	0.355	0.498	0.556	0.667	0.704	0.801	0.796	0.937	0.939	Lower arch

**Figure 4 -:**
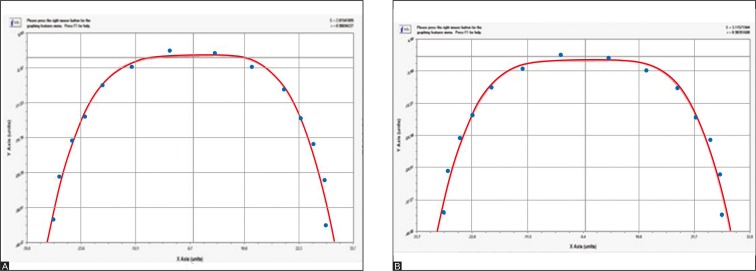
A) A sample of dental arch drawn by 4th degree polynomial, using coordinates obtained by CMM. B) A sample of maxillary arch drawn by 4th degree polynomial, using coordinates obtained by the laser scanner.

The mean difference in the coordinates recorded in the entire arch and for each tooth was 0.616 mm. These differences do not cause clinically significant changes when drawing patient's arch form (Figs 4A,B).

## DISCUSSION

In this study, a newly developed 3D laser scanner was compared with a CMM with regards to measuring the dimensions of a Teflon cube and recording the coordinates of clinical bracket points. The coordinates of clinical bracket points are helpful in drawing a polynomial curve of the dental arch. No significant differences were detected in the dimensions of the Teflon cube measured by the two devices. However, according to Dahlberg's formula, the difference between the mean values of the coordinates of clinical bracket points was found to be 0.616 mm in the X and Y coordinates when the readings of the 3D laser scanner and the reference device (CMM) were compared. This difference between the two devices may be due to the different linear measurements and due to recording only one point. For example, the linear distance between two distinct points may be nearly the same, although the exact coordinates may differ. There is also another fact that should be taken into account when digitizing CBPs by a 3D laser scanner: the difference between CBP's width (1 mm, on average) and irradiated laser beam width (100 µm or 0.1 mm, on average). Even though the software used to perform landmark digitization calculated the geometric center of each point, a difference between the center point determined by two devices may have contributed to potential differences in measurements.

This difference may also be attributed to the different spatial location of these points (due to the position of clinical bracket points in different spatial planes). The variability of this difference in different tooth series is another important issue that needs to be considered. An increasing gradient exists in the amount of this difference as moving from anterior towards posterior teeth, since the least difference was observed at central incisors and the maximum difference at molar teeth. Considering the fact that convexity of teeth increases in the dental arch from anterior towards posterior teeth (labial surface of incisors is more flat than the buccal surface of posterior teeth), it can be suggested that the difference between the two devices is due to the different placement of the CMM probe compared to the point captured by the 3D scanner on more convex surfaces in comparison to straighter surfaces. The amount of this difference was calculated separately for the maxilla and mandible. It seems that no difference exists in recording coordinates at different areas of the the mandible and the maxilla. In general, the amount of difference between the X and Y coordinates of different tooth series was slightly different and less than the clinically perceptible level, since this difference was less than 1 mm which is the human eye accuracy.

To date, several studies have assessed the accuracy of 3D methods. Nearly all of them were based on assessment and comparison between linear dimensions (such as tooth size,^20^^,^ 22 ^-^ 26 intercanine distance, interpremolar distance, intermolar distance,^24^^,^ 24 ^,^ 27 ^,^ 28 tooth crown height,^29^ and arch length^23^^,^ 24 ^,^ 28 ^,^ 30 ^,^ 31) and a reference method (for instance, manual measurement on dental casts). In the present study, we compared Descartes' coordinates of specific points. To this end, we compared the coordinates recorded by our newly designed 3D laser scanner with readings yielded by an accurate reference device (CMM). Once the spatial coordinates of specific points required by the clinician are recorded with an acceptable accuracy, linear (related to two points) and angular (related to three points) measurements will have an acceptable accuracy as well.

According to a systematic review,^32^ the mean difference between 3D techniques and reference methods in measurement of mesiodistal width of teeth was 0.01 to 0.3 mm. Also, the mean difference between 3D techniques and reference methods was 0.04 to 0.4 mm when measuring intercanine, interpremolar and intermolar distances, as well as 0.1 mm when measuring tooth crown height, and 0.19 to 0.8 mm when measuring arch length. With our laser scanner, the differences in Descartes' coordinates of clinical bracket points varied from 0.2 to 0.9 mm at various areas of the dental arch with a mean difference of 0.616 mm.

Furthermore, the reproducibility (reliability coefficient) of measurements performed by our 3D laser scanner ranged from fair to good.^33^

Accuracy of our laser scanner, especially at the anterior arch, was acceptable for clinical purposes (the overall mean difference of 0.468 mm with the area between central incisors and canine teeth used as reference). The lower accuracy of the device in recording the coordinates of points at the posterior arch is less critical considering the U-shaped form of the dental arch and the main goal of measuring these coordinates, which is to determine the clinical bracket points or drawing the arch form. A slight difference between the coordinates of these points and their actual coordinates was within the error range of our device and does not cause significant changes when drawing the arch form (Figs 4A,B).

It is suggested that the accuracy of measurements be increased in future studies by improving the rotational mechanics of the device, enhancing the accuracy of CCD imaging and using a thinner probe in the CMM.

Within the limitations of this study, the following conclusions were drawn:

The accuracy of clinical bracket point coordinates measured by our laser scanner was equal to that of CMM. The mean difference in measurements was within the range of operator errors (mean of 0.616 mm). One error in recording point coordinates by CMM is due to the operator and the width of the probe. However, this error has no clinical significance. In the laser scanner technique, error is attributed to the width of the marked point which is much wider than the width of the irradiated laser.
